# Supplementary material for: CavitySpace: A Database of Potential Ligand Binding Sites in the Human Proteome
Source: Biomolecules. 2022 Jul 11;12(7):967. doi: 10.3390/biom12070967 (PMC9312471; doi:10.3390/biom12070967)
Supplement: Supplementary file 1 [file biomolecules-12-00967-s001.zip › biomolecules-1799999-supplementary.pdf]

# Supplementary Information

## Supplementary Methods

### *CAVITY program*

We applied CAVITY program to identify cavities on the protein surface. CAVITY is a geometry-based method that employs a probe sphere and navigates the protein surface to simulate the microcosmic kinetic processes used by small molecules during their attempt to bind the protein. The detailed procedures can be found in the CAVITY paper [1].

In simple terms, CAVITY defines a potential binding cavity as "a cavity composed of one to several concave clefts which could be bound by one ligand". The program creates a box to include all the protein atoms and defines all points that are not occupied by any protein atoms as "vacant points". Then a probe sphere with a radius value (default is 10Å) rolls over the protein surface and all points the sphere touches are defined as the "outside points". The remained vacant point clusters are potential cavities. Next, a "layer depth" is defined as the distance between "vacant grid points" and the "outside points". Finally, we can define a rational cavity if the cavity is deeper than a specific depth (controlled by a parameter "minimal depth"). However, the detected binding site can be very large but the size of ligand which binds to a protein is limited, so we will try to separate a large cavity into two or more smaller cavities. As a result, we defined a parameter called "maximal joint depth" to control the separation of large cavities. In addition to the above parameter, there are some other parameters, such as the volume size of cavities that are defined to help us identify potential cavities. In summary, CAVITY used a series of geometry-based criteria to identify cavities in a protein.

CAVITY program uses a DrugScore to define the druggability of a cavity. The DrugScore is defined as:

$$DrugScore = \frac{N_B \times N_H}{N_V} - 6000 \times \frac{N_S}{N_V}$$

Where $N_B$ is the number of the hydrophobic and hydrogen-bonding grid points inside the cavity, $N_H$ is the number of hydrophobic grid points, $N_V$ is the total number of the grid points inside the cavity, and $N_S$ is the number of grid points in the lip layers of the cavity.

Based on the DrugScore, the cavities were divided into strong (DrugScore ⩾ 600), medium (-180 ⩽ DrugScore < 600) and weak (DrugScore < -180) categories. The cutoff values were defined by the NRDLD data set [2] containing crystal structures of 71 druggable and 44 less druggable proteins by literature search and data mining. More information can be found in the CAVITY paper [1].

### *Pocket comparison*

We used PocketMatch to compare binding sites for function analysis [3]. PocketMatch represents each binding site as 90 lists of sorted distances capturing the shape and chemical nature of the site and then aligns them incrementally to obtain a similarity score called PMScore, which is scaled between 0 and 1, where 1 indicates identity. PocketMatch provides two type scores, one score called PMSmax implying significant similarity in the whole site and the other score called PMSmin reflecting a local sub-structural match. We select the PMSmax to evaluate

the pocket similarity because it is believed to indicate biologically meaningful similarities.

### *Clustering*

We clustered the total 111,330 cavities with the Butina algorithm [4]. We have tried different thresholds of PMSmax. With the threshold of PMSmax ≥ 0.8, 89,467 cavities have no similar cavity, reminding that the threshold is too strict. With the threshold of PMSmax ≥ 0.7, 50,067 cavities still have no similar cavity. The remaining cavities were grouped into 12,942 clusters and 586 of them contain more than 10 cavities. When the threshold of PMSmax ≥ 0.6 was used, 11,213 cavities have no similar cavity. The remaining cavities were grouped into 8,015 clusters and 538 of them contain more than 10 cavities. When the threshold of PMSmax ≥ 0.5 was used, 983 cavities have no similar cavity and the remaining cavities were grouped into 1,864 clusters. However, the first cluster contains 31.6% of the cavities. It is obvious that the cavities cannot be classified well. Finally, we select the threshold of PMSmax ≥ 0.6 to make a clustering analysis.

## Supplementary Results

### *An example of conformation difference between AlphaFold structures and hrefPDB structures*

The hrefPDB structure of alpha-actinin-2 (UniProt ID: P35609) is the single chain A from the crystal structure of human muscle alpha-actinin-2 (PDB entry: 4D1E). Even if the AlphaFold structure and hrefPDB structure of alpha-actinin-2 have a sequence identity of 97.8%, the cavity detection results are very different (12 detected cavities for hrefPDB structure and 34 detected cavities for AlphaFold structure). The hrefPDB structure is an unfolded bundle of $\alpha$-helixes corresponding to its SR1-SR4 domains (Figure S1a) while the AlphaFold structure is a half-folded bundle of $\alpha$-helixes where SR1 binds with SR4, and SR2 binds with SR3 [5]. The difference in conformation between two structures might result from different ensemble states. Alpha-actinin-2 prefers an unfolded dimeric structure in most related PDBs including 4D1E, which have complete SR1-SR4 domains in one single chain, except for the crystal structure of sarcomeric protein FATZ-1 in complex with half dimer of alpha-actinin-2 (PDB entry: 7ANK), where one chain has SR1, SR2 domains and the other chain has SR3, SR4 domains. The structure of the 7ANK complex can be well aligned with the AlphaFold structure, which reminds us that the AlphaFold structure might learn the folding pattern from 7ANK rather than 4D1E.

Some of the excessive cavities detected in the AlphaFold structure, with strong and medium druggability, were mostly detected from the folded conformation between two bundles of $\alpha$-helixes, as shown in Figure S1b. Therefore, we can conclude that the specific conformation has a non-negligible effect on cavity detection.

### *Pocket analysis*

Cysteinyl leukotriene receptor 1 ($CysLT_1R$) is a G protein-coupled receptor as well as a key player in allergic and inflammatory disorders and zafirlukast is a selective antagonist of $CysLT_1R$ [6]. In order to find potential new binding sites for zafirlukast, we investigated the cavity cluster that the zafirlukast binding site belongs to and screened all cavities with PMSmax > 0.8 that have strong druggability and do not have known PDB structures. Among 16 compliant AlphaFold cavities, we chose only one representative cavity for those cavities that were in the same domain or motif, such as the seven-transmembrane domain of GPCR and

kelch motif. In addition, we abandoned cavities from Cytochrome P450. At last, we obtained 7 representative AlphaFold cavities. We performed molecular docking between target proteins and zafirlukast using AutoDock Vina 1.2 [7] (Table S2). Docking study showed that zafirlukast can bind to these cavities with high affinity, which can be experimentally tested in the future.

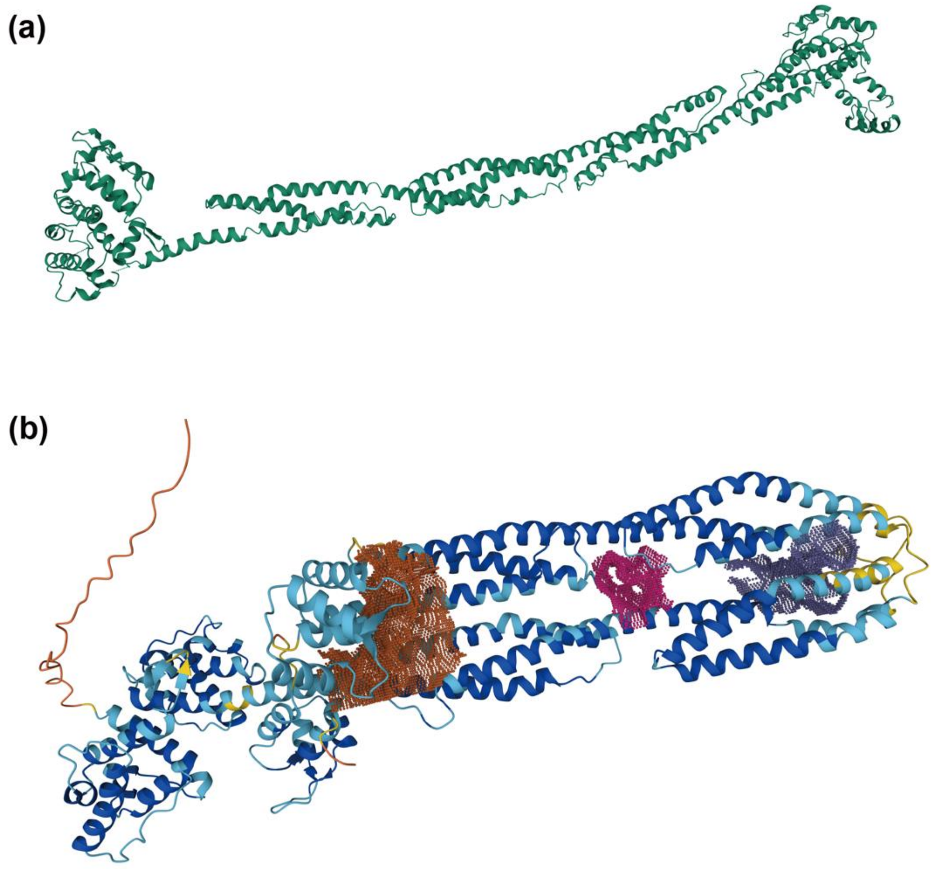


**Figure S1.** An example of conformation difference between AlphaFold structures and hrefPDB structures. (a) The hrefPDB structure of alpha-actinin-2 (4D1E_A). (b) The cavities with strong (brown dots) and medium (purple dots and blue dots) druggability detected from the folded conformation of the AlphaFold structure (P35609) cannot be detected in the hrefPDB structure.

**Table S1.** Databases of protein pockets since 2004.

| Database | Publication Year | Description | Reference |
|---|---|---|---|
| **ProBiS-Dock Database** | 2021 | predicted small molecule and cofactor binding sites | [8] |
| **HKPocket** | 2019 | predicted human kinase pocket | [9] |
| **PocketDB** | 2018 | predicted small-molecule binding pockets | [10] |
| **TuberQ** | 2014 | *Mycobacterium tuberculosis* protein druggability | [11] |
| **sc-PDB** | 2014 | predicted ligandable binding sites | [12] |
| **KLIFS** | 2014 | kinase–ligand interaction fingerprints and structure | [13] |
| **Bival-bind** | 2014 | protein complexes with multivalent binding ability | [14] |
| **FireDB** | 2013 | catalytic and biologically relevant small ligand-binding residues from PDB | [15] |
| **Pocketome** | 2011 | experimentally solved conformational ensembles of druggable binding sites in proteins | [16] |
| **PoSSuM** | 2011 | similar protein–ligand binding and putative pockets | [17] |
| **fPOP** | 2009 | protein functional surfaces identified by analyzing the shapes of binding sites in both holo and apo forms | [18] |
| **CREDO** | 2009 | protein–ligand interactions with structural interaction fingerprints and novel features | [19] |
| **SuperSite** | 2008 | metabolite and drug binding sites in proteins | [20] |
| **LigASite** | 2007 | biologically relevant binding sites in proteins with known apo-structures | [21] |
| **SitesBase** | 2006 | structure-based protein–ligand binding site comparisons | [22] |
| **PDBSite** | 2005 | protein active sites and their spatial environment | [23] |
| **Het-PDB Navi2** | 2004 | protein–small molecule interactions | [24] |

**Table S2.** Seven representative AlphaFold cavities that are similar to the zafirlukast binding site in cysteinyl leukotriene receptor 1.

| UniProt ID | Protein Name | Cavity ID | PMSmax | Vina Score (kcal/mol) |
|---|---|---|---|---|
| Q8TDU9 | Relaxin-3 receptor 2 | 1 | 0.849 | -9.80 |
| Q9UL12 | Sarcosine dehydrogenase, mitochondrial | 5 | 0.835 | -10.81 |
| Q96S06 | Lipase maturation factor 1 | 1 | 0.826 | -9.35 |
| Q12887 | Protoheme IX farnesyltransferase, mitochondrial | 2 | 0.822 | -10.60 |
| P06133 | UDP-glucuronosyltransferase 2B4 | 1 | 0.810 | -12.15 |
| Q9H568 | Actin-like protein 8 | 1 | 0.806 | -11.56 |
| Q9H270 | Vacuolar protein sorting-associated protein 11 homolog | 3 | 0.805 | -10.31 |

**References**


1. Yuan YX, Pei JF, and Lai LH. Binding site detection and druggability prediction of protein targets for structure-based drug design. *Curr. Pharm. Des.* 2013; ***19***(12): 2326-2333.
2. Krasowski A, Muthas D, Sarkar A, et al. DrugPred: A structure-based approach to predict protein druggability developed using an extensive nonredundant data set. *J. Chem. Inf. Model.* 2011; ***51***(11): 2829-2842.
3. Yeturu K and Chandra N. PocketMatch: A new algorithm to compare binding sites in protein structures. *BMC Bioinform.* 2008; ***9***(1): 1-17.
4. Butina D. Unsupervised data base clustering based on Daylight's fingerprint and Tanimoto similarity: A fast and automated way To cluster small and large data sets. *J. Chem. Inf. Comput. Sci.* 1999; ***39***(4): 747-750.
5. Ribeiro EdA, Pinotsis N, Ghisleni A, et al. The structure and regulation of human muscle $\alpha$-actinin. *Cell* 2014; ***159***(6): 1447-1460.
6. Luginina A, Gusach A, Marin E, et al. Structure-based mechanism of cysteinyl leukotriene receptor inhibition by antiasthmatic drugs. *Sci. Adv.* 2019; ***5***(10): eaax2518.
7. Eberhardt J, Santos-Martins D, Tillack AF, et al. AutoDock Vina 1.2.0: New docking methods, expanded force field, and Python bindings. *J. Chem. Inf. Model.* 2021; ***61***(8): 3891-3898.
8. Konc J, Lešnik S, Škrlj B, et al. ProBiS-Dock database: A web server and interactive web repository of small ligand–protein binding sites for drug design. *J. Chem. Inf. Model.* 2021; ***61***(8): 4097-4107.
9. Wang H, Qiu J, Liu H, et al. HKPocket: Human kinase pocket database for drug design. *BMC Bioinform.* 2019; ***20***(1): 1-11.
10. Bhagavat R, Sankar S, Srinivasan N, et al. An augmented pocketome: Detection and analysis of small-molecule binding pockets in proteins of known 3D structure. *Structure* 2018; ***26***(3): 499-512 e2.
11. Radusky L, Defelipe LA, Lanzarotti E, et al. TuberQ: A Mycobacterium tuberculosis protein druggability database. *Database* 2014; ***2014***: bau035.
12. Desaphy J, Bret G, Rognan D, et al. sc-PDB: A 3D-database of ligandable binding sites—10 years on. *Nucleic Acids Res.* 2015; ***43***(D1): D399-D404.
13. van Linden OPJ, Kooistra AJ, Leurs R, et al. KLIFS: A knowledge-based structural database to navigate Kinase–ligand interaction space. *J. Med. Chem.* 2014; ***57***(2): 249-277.
14. Meyer T and Knapp E-W. Database of protein complexes with multivalent binding ability: Bival-bind. *Proteins* 2014; ***82***(5): 744-751.
15. Maietta P, Lopez G, Carro A, et al. FireDB: A compendium of biological and pharmacologically relevant ligands. *Nucleic Acids Res.* 2013; ***42***(D1): D267-D272.
16. Kufareva I, Ilatovskiy AV, and Abagyan R. Pocketome: An encyclopedia of small-molecule binding sites in 4D. *Nucleic Acids Res.* 2011; ***40***(D1): D535-D540.
17. Ito J-I, Tabei Y, Shimizu K, et al. PoSSuM: A database of similar protein–ligand binding and putative pockets. *Nucleic Acids Res.* 2011; ***40***(D1): D541-D548.
18. Tseng YY, Chen ZJ, and Li W-H. fPOP: Footprinting functional pockets of proteins by comparative spatial patterns. *Nucleic Acids Res.* 2009; ***38***(suppl_1): D288-D295.
19. Schreyer A and Blundell T. CREDO: A protein–ligand interaction database for drug discovery. *Chem. Biol. Drug. Des.* 2009; ***73***(2): 157-167.

20. Bauer RA, Günther S, Jansen D, et al. SuperSite: Dictionary of metabolite and drug binding sites in proteins. *Nucleic Acids Res.* 2008; ***37***(suppl_1): D195-D200.
21. Dessailly BH, Lensink MF, Orengo CA, et al. LigASite—a database of biologically relevant binding sites in proteins with known apo -structures. *Nucleic Acids Res.* 2007; ***36***(suppl_1): D667-D673.
22. Gold ND and Jackson RM. SitesBase: A database for structure-based protein–ligand binding site comparisons. *Nucleic Acids Res.* 2006; ***34***(suppl_1): D231-D234.
23. Ivanisenko VA, Pintus SS, Grigorovich DA, et al. PDBSite: A database of the 3D structure of protein functional sites. *Nucleic Acids Res.* 2005; ***33***(suppl_1): D183-D187.
24. Yamaguchi A, Iida K, Matsui N, et al. Het-PDB Navi.: A database for protein–small molecule interactions. *J. Biochem.* 2004; ***135***(1): 79-84.
